# Ultraprocessed Foods: Effect on Physical Performance of Elderly with Cognitive Impairment

**DOI:** 10.1002/alz70860_104376

**Published:** 2025-12-23

**Authors:** Marcelle Ferreira Saldanha, João Marcos Silva Borges, Lorrayne F Barreto Rocha Faria Martins, Leonardo Ryuiti Kimoto, Julia Cardoso Costa Cardoso Costa, Giovanna Correia Pereira Moro, Gabriela Tomé Oliveira Engelmann, Marco Aurelio Romano‐Silva, Jonas Jardim de Paula, Bernardo de Mattos Viana, Ann Kristine Jansen, Maria Aparecida Camargos Bicalho

**Affiliations:** ^1^ Cog‐Aging Research Group, Belo Horizonte, Minas Gerais, Brazil; ^2^ Sciences Applied to Adult Health Postgraduate Program, School of Medicine, Universidade Federal de Minas Gerais (UFMG), Belo Horizonte, Minas Gerais, Brazil; ^3^ Cog‐Aging Group Research, Brazil, Belo Horizonte, Minas Gerais, Brazil; ^4^ Federal University of Minas Gerais, Belo Horizonte, Minas Gerais, Brazil; ^5^ Cog‐Aging Research Group, Universidade Federal de Minas Gerais (UFMG), Belo Horizonte, Minas Gerais, Brazil; ^6^ Universidade Federal de Minas Gerais, Belo Horizonte, Minas Gerais, Brazil; ^7^ Cog Aging Research Group, Brazil, Belo Horizonte, Minas Gerais, Brazil; ^8^ Undergraduate Medicine, Faculty of Medicine, Universidade Federal de Minas Gerais (UFMG), Belo Horizonte, Minas Gerais, Brazil; ^9^ Older Adult's Psychiatry and Psychology Extension Program (PROEPSI), School of Medicine, Universidade Federal de Minas Gerais (UFMG), Belo Horizonte, Minas Gerais, Brazil; ^10^ Universidade Federal de Minas Gerais, Belo Horizonte, Brazil; ^11^ Jenny de Andrade Faria Institute – Outpatient Reference Center for the Elderly, Universidade Federal de Minas Gerais (UFMG), Belo Horizonte, Minas Gerais, Brazil; ^12^ Molecular Medicine Program, School of Medicine, Federal University of Minas Gerais, Belo Horizonte, Minas Gerais, Brazil; ^13^ Molecular Medicine Postgraduate Program, School of Medicine, Universidade Federal de Minas Gerais (UFMG), Belo Horizonte, Minas Gerais, Brazil; ^14^ Neurotec R National Institute of Science and Technology (INCT‐Neurotec R), Faculty of Medicine, Federal University of Minas Gerais, Belo Horizonte, Minas Gerais, Brazil; ^15^ Department of Psychiatry, School of Medicine, Federal University of Minas Gerais, Belo Horizonte, Minas Gerais, Brazil; ^16^ National Institute of Science and Technology Neurotec R (INCT‐MM), Belo Horizonte, Minas Gerais, Brazil; ^17^ Older Adult's Psychiatry and Psychology Extension Program I Federal University of Minas Gerais, Belo Horizonte, MG, Brazil; ^18^ Neurotec R National Institute of Science and Technology (INCT‐Neurotec R), Faculty of Medicine, Universidade Federal de Minas Gerais (UFMG), Belo Horizonte, Minas Gerais, Brazil; ^19^ National Institute of Science and Technology (INCT‐Neurotec R), Faculty of Medicine, Federal University of Minas Gerais, Belo Horizonte, Minas Gerais, Brazil; ^20^ Older Adult's Psychiatry and Psychology Extension Program Federal University of Minas Gerais, Belo Horizonte, Minas Gerais, Brazil; ^21^ Geriatrics and Gerontology Center Clinical Hospital of University of Minas Gerais, Belo Horizonte, Minas Gerais, Brazil; ^22^ Jenny de Andrade Faria Institute – Outpatient Reference Center for the Elderly, Universidade Federal de Minas Gerais (UFMG), Belo Horizonte, Minas Gerais, Brazil; ^23^ Hospital das Clínicas da UFMG, University Hospital, Universidade Federal de Minas Gerais (UFMG), Belo Horizonte, Minas Gerais, Brazil; ^24^ Graduate Program in Applied Sciences to Adult Health, Faculdade de Medicina, Universidade Federal de Minas Gerais, Belo Horizonte, Brazil; ^25^ Graduate Program in Molecular Medicine, Belo Horizonte, Minas Gerais, Brazil; ^26^ UFMG, Belo Horizonte, Brazil; ^27^ INCT – NeuroTecR and CTMM, Belo Horizonte, Minas Gerais, Brazil; ^28^ Department of Internal Medicine, School of Medicine, Federal University of Minas gerais, Belo Horizonte, Minas Gerais, Brazil

## Abstract

**Background:**

The consumption of ultraprocessed foods (UPFs) is related to cognitive decline. In Brazil, about 20% of daily calories come from UPFs. However, there are few studies on the consumption of these foods in elderly individuals with cognitive decline. Objective: To assess the consumption of UPFs in elderly individuals with cognitive impairment, to identify the main foods related to this consumption, and to explore its association with anthropometric parameters, body composition and sarcopenia.

**Method:**

A cross‐sectional study was conducted with elderly participants from the Cog‐Aging cohort study, diagnosed with mild cognitive impairment or dementia. Anthropometric measurements, body composition tests, handgrip strength (HGS) tests, and physical performance (timed up and go test ‐ TUG) were performed. Total UPF consumption was assessed using a food frequency questionnaire, stratified into tertiles. Kruskal–Wallis test with Dunn's post‐hoc analysis was used to compare the variables between the UPF consumption groups divided in tertiles.

**Result:**

Seventy‐five elderly individuals were evaluated, 20 with dementia and 55 with MCI, with a mean age of 77.6 years and an average education level of 6.8 years (SD ± 5.43). The median weight was 66.0 kg, 33% were classified as eutrophic and 21,3% as obese, according to BMI. The median daily UPF consumption was 129.9 g. Foods such as mayonnaise, frozen potatoes, ice cream, popsicles, candies, sugary beverages, and cereals were the main contributors to high UPF consumption. There was no association between UPF consumption, anthropometry, body composition, and HGS. TUG test values (in seconds) were statistically different: 11.13 (1st tertile), 11.18 (2nd tertile), and 15.0 (3rd tertile), with the worst performance associated with the highest tertile of UPF consumption group (*p* = 0.03).

**Conclusion:**

The consumption of UPFs in elderly individuals with cognitive impairment was high, and the main foods contributing to this consumption were mayonnaise, frozen potatoes, ice cream, popsicles, candies, gelatin, cookies, instant noodles, and sugary beverages. Additionally, high UPF consumption was associated with worse physical performance.

